# High prevalence of clarithromycin resistance and effect on Helicobacter pylori eradication in a population from Santiago, Chile: cohort study and meta-analysis

**DOI:** 10.1038/s41598-019-56399-7

**Published:** 2019-12-27

**Authors:** A. Arenas, C. Serrano, L. Quiñones, P. Harris, M. Sandoval, M. Lavanderos, R. Sepúlveda, S. Maquilón, A. Echeverría, C. Ríos, E. Fuentes-López, L. Rojas, A. Jorquera, M. Pizarro, M. C. Camargo, A. Riquelme

**Affiliations:** 1 Departamento de Gastroenterología, Escuela de Medicina, Pontificia Universidad Católica de Chile, Santiago, Chile; 2 Departamento de Gastroenterología y Nutrición pediátrica, Pontificia Universidad Católica de Chile, Santiago, Chile; 3 Laboratorio CQF, Departamento de Oncología Básico-Clínica, Facultad de Medicina, Universidad de Chile, Santiago, Chile; 4 Escuela de Medicina, Pontificia Universidad Católica de Chile, Santiago, Chile; 5 Departamento de Medicina Interna, Escuela de Medicina. Pontificia Universidad Católica de Chile, Santiago, Chile; 6 Servicio de Endoscopia, Hospital de Curanilahue, Curanilahue, Chile; 7 Division of Cancer Epidemiology and Genetics, National Cancer Institute, Bethesda, USA; 8 Departamento de Medicina Interna, Universidad de la Frontera, Frontera, Chile; 9 Departamento de Ciencias de la Salud, Facultad de Medicina. Pontificia Universidad Católica de Chile, Santiago, Chile

**Keywords:** Bacterial genes, Stomach diseases

## Abstract

*Helicobacter pylori* (*H. pylori)* eradication using standard triple therapy (STT) with proton pump inhibitors (PPI), amoxicillin and clarithromycin (CLA) has been the standard in Latin America. However, CLA resistance is a rising problem affecting eradication rates. Genetic polymorphisms of *CYP2C19*, a PPI metabolizer may also affect eradication. The primary aims of this study were to evaluate the effect of clarithromycin resistance on *H. pylori* eradication in a population from Santiago, and to establish the pooled clarithromycin resistance in Santiago, Chile. Symptomatic adult patients attending a tertiary hospital in Santiago were recruited for this study. CLA resistance and the polymorphisms of CYP2C19 were determined on DNA extracted from gastric biopsies, using PCR. The STT was indicated for 14 days and eradication was determined by a urea breath test 4–6 weeks after therapy. A meta-analysis of CLA resistance studies among adult residents in Santiago was performed. Seventy-three out of 121 consecutive patients had positive rapid urease test (RUT) and received STT. Sixty-nine patients (95%) completed the study. The *H. pylori* eradication rate was 63% and the prevalence of CLA resistance was 26%. According to the *CYP2C19* polymorphisms, 79.5% of the RUT-positive patients were extensive metabolizers. Multivariable analyses showed that only CLA resistance was significantly and inversely associated with failure of eradication (OR: 0.13; 95% confidence interval [95% CI], 0.04–0.49). A meta-analysis of two previous studies and our sample set (combined n = 194) yielded to a pooled prevalence of CLA resistance of 31.3% (95% CI 23.9–38.7). Our study shows that CLA resistance is associated with failure of *H. pylori* eradication. Given the high pooled prevalence of CLA resistance, consideration of CLA free therapies in Santiago is warranted. We could recommend bismuth quadruple therapy or high-dose dual therapy, according to bismuth availability. Further studies need to evaluate the best therapy.

## Introduction

*Helicobacter pylori (H. pylori)* infection affects approximately 50% of the population worldwide. Prevalence in developing countries ranges between 70% to 90%^1,2^. In Chile, more than 70% of adults are infected by this bacterium^3^. *H. pylori* infection plays an important role in the development of duodenal ulcers, gastric cancer (GC) and mucosa associated lymphoid tissue lymphoma. The International Agency for Research on Cancer classified *H. pylori* as one of the primary risk factors for the development of noncardia GC^4^. In Chile, GC is the first cause of cancer death for men and the third in women^5,6^.

*H. pylori* eradication reduces the risk of development of the above mentioned clinical outcomes. Standard triple therapy (STT) with clarithromycin (CLA) and amoxicillin for 7 to 14 days is recommended as treatment for low CLA resistance regions with expected eradication success rates up to 85%^7^. The standard of care in Chile, as recommended by nationwide clinical guidelines is triple therapy with CLA^8^.

During the last decades, an increase in CLA resistance rates and a parallel decrease in eradication efficacy has been observed globally^9^. CLA is a bacteriostatic antibiotic that binds in a reversible manner to the peptidyl transferase located in dominion V of the 23S rRNA gene, inhibiting protein synthesis in *H. pylori*^10^. Single nucleotide mutations in A2142G and A2143G positions are the most common variations described^11–13^. A systematic review of Latin American studies by Camargo *et al*., reported a 12% pooled prevalence of resistance for CLA, 53% for metronidazole, 4% for amoxicillin, 6% for tetracycline, 15% for fluoroquinolones and 8% for dual CLA and metronidazole^14^. In two recently studies, Hooi JKY *et al*. and Savoldi A *et al*., analysed the information available worldwide describing an increasing antibiotic resistance in most regions. Resistance rates to CLA, metronidazole, and levofloxacin were ≥15%, which have a great effect on efficacy of CLA-containing regimens^9,15,16^.

In Chile, recent studies showed an increasing frequency of CLA resistance over 20%^17–19^. The Latin American, Toronto and Maastricht V consensus recommend not to use STT in regions with CLA resistance rates >15%. Alternatively, they recommend CLA-free regimes such as bismuth quadruple therapy (BQT)^7,20–22^. According to the meta-analysis by Fischbach *et al*., eradication success is 66% in the presence of CLA resistance^23^.

Proton pump inhibitors (PPIs) are used to maintain an alkaline pH and avoid antibiotic inactivation, particularly for CLA. In addition, elevated pH drives *H. pylori* into a replicative state contributing to increased antibiotic sensitivity^24^. The main isoenzyme involved in PPI metabolism is P4502C19 cytochrome (CYP2C19). The *CYP2C19* gene is extensively polymorphic with 34 allelic variants composed of Single Nucleotide Polymorphisms (SNPs). The main allelic variants described are *2, *3 and *17^25^. CYP2C19*2 is an allele that is produced by substitution of a single base (rs4244285, 681G <A) in exon 5 causing a change into the alternative splicing site dramatically reducing drug metabolism. Defective allele CYP2C19*3 (rs4986893, 636G >A) produces a stop codon generating an abnormal form of the enzyme. Finally, variant *17 (rs12248560, −806C >T), increases enzymatic activity driving expression to an ultra-rapid metabolism^25^.

Several phenotypes have been identified for CYP2C19. The extensive or normal metabolizer (EM) characterized by wild type homozygous alleles *1/*1 or *1/*17 haplotype; the intermediate metabolizer (IM) carriers of *1/*2, *1/*3 and *2/*17 haplotypes; the poor metabolizer (PM) characterized by *2/*2, *2/*3, *3/*3 haplotypes and the ultra-fast metabolizer (UM) which is a *17 (*17/*17) homozygous carrier. Phenotypes EM and UM metabolize PPIs at a fast rate, so higher doses of these agents are required to achieve the same effectiveness that in IM and PM phenotypes^26^.

*CYP2C19 p*olymorphisms show high population variability. Eradication rates vary according to the metabolizer phenotype. Higher eradication rates are achieved in PM (80%) in comparison to EM (60%)^27,28^. However, therapies that use PPIs such as rabeprazole and esomeprazole show less sensitivity CYP2C19 genetic polymorphisms^29^.

The primary aims of this study were: (i) to evaluate the effect of CLA resistance on *H. pylori* eradication success with STT based on Omeprazole-Amoxicillin-CLA for 14 days in a population from Santiago, (ii) to conduct a meta-analysis of the CLA resistance studies to calculate the pooled prevalence of CLA resistance in adult residents in Santiago, Chile. The secondary aims were: (i) to determine the effect of phenotypes of *CYP2C19* polymorphisms on *H. pylori* eradication, (ii) to determine the *H. pylori* eradication rate using CLA-free quadruple therapy with Esomeprazole-Tetracycline-Metronidazole-Bismuth (ETMB) for 14 days as a second line treatment.

## Methods

### Study design

Prospective cohort study, we consecutively recruited symptomatic adult patients (18–75 years old), who had requested an endoscopy by their attending physician, between June 2017 and February 2018. Recruitment was performed in two endoscopic centers of the Healthcare network (Red de Salud UC-CHRISTUS) at the Pontificia Universidad Católica in Santiago. All patients signed an informed consent prior to upper gastrointestinal endoscopy. The project was approved by the Ethics Committee, School of Medicine, Pontificia Universidad Católica de Chile (project ID: 161205012) and was conducted according to the Helsinki declaration and Good Clinical Practices.

All patients completed a sociodemographic and clinical questionnaire. Exclusion criteria included individuals with partial gastrectomy for GC, GC treated by endoscopic resection, bariatric surgery, recent digestive bleeding, pregnancy or nursing period, PPI use for 7 days before endoscopy, antibiotic use 4 weeks before endoscopy, previous *H. pylori* eradication treatment and previous known intolerance or allergic reaction to the antibiotics used, and other malignancies.

### *H. pylori* diagnosis and treatment

Rapid urease test (RUT) (Gastrex, Gilly les Citeaux, France) was performed during endoscopy to all patients. Positivity within 30 minutes resulted in an *H. pylori* diagnosis. RUT positive patients received STT consisting of 500 mg of CLA, 1 gr of amoxicillin and 20 mg of omeprazole every 12 hours for 14 days. RUT negative patients did not receive any antibiotic treatment and were excluded from the analysis. Follow-up for adverse effects was performed via telephone 7 days after treatment. Four to six weeks following the completion of the treatment. Urease breath test (UBT) (Heliforce, Beijing, China) was performed 4–6 weeks post therapy to determine eradication efficacy.

### CLA resistance

Single nucleotide mutations in 23S rRNA gene (A2142G and A2143G) were determined to infer *H. pylori* resistance to CLA. DNA was extracted from four antral mucosal biospies obtained during endoscopy, using the QIAamp DNA mini kit (QIAGEN, Hilden, Germany) according to manufacturer instructions. PCR based amplification of 23S rRNA gene was performed using GoTaq Flexi DNA Polymerase (Promega, Madison, WI) in a PTC-100 Thermal Cycler (MJ Research, Waltham, MA). 23S-rRNA gene mutations associated with CLA resistance were determined as previously described^24^. Briefly, 10 μL of 23S-rRNA PCR products (267-bp) were digested with the restriction enzymes *Bbs*I (5 U), or *Bsa*I (5 U) (New England Biolabs, Ipswich, MA) in a final volume of 20 μL, according to the manufacturer’s instructions. Both 23S-rRNA amplicons and digested products were separated on 1.5% or 3% agarose gels respectively (SeaKem LE agarose; Lonza, Rockland, ME). DNA samples extracted from *H. pylori* strains (n = 2) with CLA point mutation A2142G and A2143G were used as internal positive controls.

### Genetic polymorphisms of the *CYP2C19* gene

Genotyping was performed from the DNA extracted from the above mentioned four gastric tissue samples using TaqMan assays from the Drug Metabolism Genotyping Assay (Thermofisher Scientific, USA) to detect *2 (c.681G >A; rs 4244285), *3 (c.636G >A; rs 4986893) and *17 (−806C >T; rs12248560) variants.

### Second line treatment

In those patients who did not achieve *H. pylori* eradication with STT, ETMB was used as a second line treatment a quadruple therapy consisting in bismuth subsalicylate (262 mg every 6 hours), Tetracycline (500 mg every 6 hours), Metronidazole (500 mg every 6 hours) and Esomeprazole (20 mg every 6 hours) for 14 days. Successful *H. pylori* eradication was confirmed by UBT, 4–6 weeks post therapy.

### Statistical analysis

For categorical variables, we compared relative frequencies using a Fisher’s exact test or chi-squared test. Continuous variables where analyzed using t-Test. Univariate logistic regression models were built to assess the association between *H. pylori* eradication and: CLA resistance, phenotypes of *CYP2C19* polymorphisms, and demographic variables (age and gender). A multivariate logistic regression model was applied to test for the association of *H. pylori* eradication with CLA resistance and phenotypes of *CYP2C19* polymorphisms, adjusting for demographic variables.

For all analyses, a two-sided statistical significance was considered at 5% level. All statistical analyses were performed in STATA 16 (StataCorp. LP, College Station, TX).

## Sample size

The sample size was calculated considering the proportion of CLA resistance described in a previous similar study by Garrido L., *et al*.^16^. Thus, using this proportion (20%) with an alpha level = 0.05, and a precision of 0.1 (half width of confidence interval), the sample size required was 62 participants. Considering an eventual loss of 10%, we proposed to recruit 69 people. In addition, the number of events per variable rule was considered to calculate the sample size necessary to perform the multivariate logistic model^30^. Simulation studies^31^ have established that for logistic regression models to analyze dichotomous data, it is recommended to include one predictor variable per 10 events in the sample (i.e. *H. pylori* eradication success). Thus, in this current study, in order to include four variables in the multivariate logistic model (CLA resistance, phenotypes of *CYP2C19* polymorphisms, age and gender), it was necessary to have 40 events. The final sample was comprised by 44 events (i.e. patients who eradicated *H. pylori* successfully).

### Meta-analysis

#### Literature search and clinical eligibility criteria

Two reviewers independently searched in the following electronic databases: databases PubMed (United States National Library of Medicine, Bethesda, MD), Gastroenterología Latinoamericana Journal; http://www.gastrolat.org) and SciELO (Scientific Electronic Library Online; http://www.scielo.org), which conduct searches for systematic reviews in several other databases following PRISMA statement^32^. To identify studies in PubMed, the following search strategy was used: “*Helicobacter pylori*” [Mesh] AND “Clarithromycin” [Mesh] AND “Resistance” [Mesh] AND Chile.

The following information was abstracted from each selected article: first author, year of publication, study location (city), year of sample collection, participant age (range or mean), number of patients, indication for endoscopic examination, prevalence (% and confidence interval) of CLA resistance, and method of resistance assessment.

The following criteria were used to exclude publications: Children population; non-Chilean (i.e., other Latin American population), studies published before 2010, duplicated sample, populations from regions outside of Santiago, and review papers. There was no language restriction on publications. Discordance about study inclusion between the two reviewers was resolved through discussion until 100% agreement was reached on the final interpretation of the data.

#### Outcome measure

The included outcome in the analysis was prevalence (% and confidence interval) of CLA resistance.

#### Assessment of risk of bias in included studies

Risk of bias in the included studies was assessed by two independent reviewers using the Scale Newcastle Ottawa.

#### Data extraction and analysis

Data extraction and analysis was performed by two independent reviewers. To calculate a pooled prevalence of CLA resistance, we performed a random effects meta-analysis of proportions using the *metaprop* command developed by Nyaga *et al*.^33^ and the *meta* command incorporated in the STATA software version 16 (StataCorp. LP, College Station, TX). Between-study heterogeneity was measured by *I*2 which (i.e., percentage of the variability in effect estimates that it is due to heterogeneity rather than sampling error), and *Cochran’s Q* statistics (provides a method for testing the differences between three or more sets of proportions). Also, a sensitivity analysis was performed by exploring the variation in proportion estimates for different values of the between study heterogeneity *I*2 statistic.

## Results

### Sociodemographic, clinical and endoscopic variables

One hundred twenty-one patients were recruited for this study, mean age 47.2 ± 13.7 years, 60% were females and 60% (n = 73) were RUT positive. Tobacco use was reported by 29% of patients, and family history of GC by 12.3%.

### Follow up and adverse effects

During the follow-up, four of the 73 RUT positive patients discontinued their participation in the study because did not accept to adhere to the treatment. Of the 69 remaining patients that initiated STT, only two interrupted treatment before 7 days because of intolerance to the treatment. We performed UBT regardless of that observation. In the telephonic interview, 80% of patients referred at least one adverse event during therapy. The main referred symptoms included altered taste (48%), followed by abdominal pain (40.6%), diarrhea (34.8%), nausea (20.3), and vomiting (5.8%). No serious adverse effects were reported.

### CLA resistance prevalence

Nineteen (26%) of the 69 RUT positive patients were CLA resistant. The predominant mutation was A2143G with 17 (89.5%) patients, while A2142G was found in only 2 (10.5%) patients.

### Polymorphism of the *CYP2C19* gene

We obtained the following genotype frequencies: 64.4% of *1/*1, 11% of *1/*2, 4.1% of *2/*17: 16.4% of *1/*17 and 4.1% of *17/*17. We analyzed metabolizer phenotypes frequencies and found 79.5% (*1/*1, *1/*17) of EM, 16.4% of IM, 0% of PM (*2/*2, *3/*3, *2/*3) and 4.1% of UM (*17/*17).

### Analysis of eradication rates

From the 69 treated RUT positive patients, 44 (63.8%) eradicated *H. pylori*. We analyzed the variables associated with *H. pylori* eradication, only CLA resistance was significant (p < 0.001). No clinical variables were associated (Table 1).

**Table 1:** Demographic and clinical characteristics by *H. pylori* eradication groups (n = 69^a^).

Variable	Eradication success n = 44	Eradication failure n = 25	*p-value*
Age, mean (±SD)	46.4 (±14.0)	49.2 (±14.1)	0.429
Gender female	26 (59%)	18 (72%)	0.284
University education	31 (70.5%)	20 (80%)	0.385
Alcohol consumption	13 (29.5%)	4 (16%)	0.256
Tobacco use	11 (25%)	4 (16%)	0.290
Pyrosis (+)	14 (31.8%)	10 (40%)	0.493
Dyspepsia (+)	22 (50%)	13 (52%)	0.873
Epigastralgia (+)	17 (38.6%)	11 (44%)	0.663
Esophagus findings	6 (13.6%)	6 (24%)	0.275
Gastric fundus findings: Congestion (+)	14 (31.8%)	9 (36%)	0.723
Gastric corpus findings: Congestion (+)	20 (45.5%)	10 (40%)	0.660
Antral findings: Congestion and/or nodular gastropathy (+)^b^	18 (40.9%)	13 (52%)	0.373
Duodenal findings: Erosions and ulcers (+)	12 (27.3%)	6 (24%)	0.766
CLA resistance	5 (11.4%)	13 (52.0%)	<0.001
Phenotype EM	34 (61.8%)	21 (38.2%)	0.235
Phenotype UM	1 (33.3%)	2 (66.7%)	
Phenotype IM	9 (81.8%)	2 (18.2%)	

^a^During the follow-up four patients dropped their participation in the study.

^b^Nodular gastropathy and congestion were pulled together for analysis. All nodular patients were *H. pylori* positive by rapid urease test.

In a multivariable analysis, CLA resistance was  inversely and significantly associated with failure of eradication (OR 0.13; 95% CI, 0.04–0.49) while CYP2C19 phenotypes were not associated (Phenotype UM: OR 0.41, 95% CI 0.02–7.83; Phenotype IM: OR 1.65, 95% CI, 0.28–9.71) (Table 2).

**Table 2:** Univariate and multivariable analyses associated to *H. pylori* eradication.

Variable	Univariate OR (95% confidence interval)	Multivariable OR^b^ (95% confidence interval)
CLA resistance	0.12 (0.04–0.40)	0.13 (0.04–0.49)
Phenotype EM^a^	1.0 (Referent)	1.0 (Referent)
Phenotype UM^a^	0.31 (0.03–3.62)	0.41 (0.02–7.83)
Phenotype IM^a^	2.78 (0.55–14.13)	1.65 (0.28–9.71)
Age	0.99 (0.95–1.02)	1.00 (0.96–1.05)
Gender female	0.56 (0.19–1.62)	0.49 (0.14–1.65)

^a^Phenotypes of *CYP2C19* polymorphisms.

^b^Age, sex, resistance to CLA, UM and EM phenotypes were included in the same model.

### Second line quadruple therapy with bismuth

Twenty of the 25 patients (80%) who did not eradicate *H. pylori* by STT, received a second line of treatment. Seventeen (85%) patients eradicated *H. pylori* with ETMB. In the telephonic interview, 86% of patients referred at least one adverse event during therapy. Adverse effects reported were nausea (70%), abdominal pain (40%), vomiting (15%), muscular weakness (15%), diarrhea (10%), and taste alterations (10%). Only one case of tongue black pigmentation related to bismuth use was reported. No serious adverse effects were reported.

#### Meta-analysis

**Figure 1:**
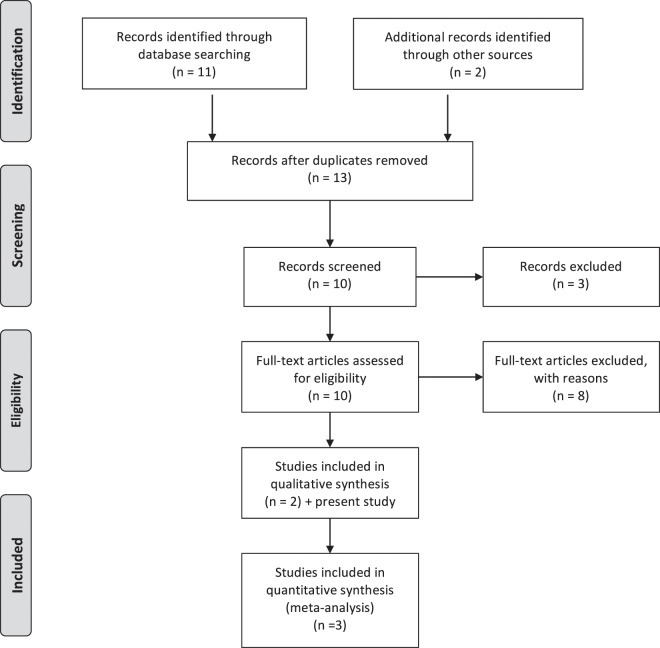
PRISMA flow diagram.

**Figure 2:**
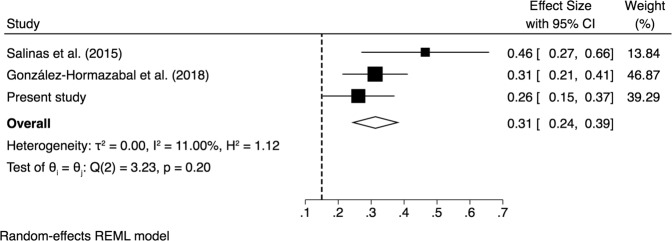
Forest plot of the proportion of CLA resistance from studies conducted in adult populations, Santiago, Chile. Symbols: ■ single studies included in the meta – analysis; − confidence interval (CI); ◇  overall pool estimated; and a reference dashed vertical line was added at 0.15 (15%) to represent the value recommended by the consensus of Maastricht V/Florence on the management of *H. pylori*.

The literature searches identified a total of 11 studies and one meeting abstract^17^. Following exclusion criteria, nine studies were excluded^14,16,19,34–40^. The specific reasons were: Pediatric population^19^; Other Latin American population^14,34,35^; study published before 2010^16,37–39^; reviews^14,35,38^; duplicated sample and population from regions outside Santiago^36,37,39,40^. Two studies met the inclusion criteria^17,18^ and were analyzed with our study, the combined sample size was 194 patients. PRISMA flow diagram is shown in Fig. 1. The characteristics of the studies are shown in Table 3. The pooled prevalence CLA resistance was 31.3% (95% CI 23.9–38.7) (Fig. 2). Heterogeneity was low (I^2^ = 11%; Q = Chi^2^ = 3.23; p = 0.20), and sensitivity analysis showed that there were minimal variations in proportion estimates for different values of the between study heterogeneity statistic *I*^2^. The overall risk of bias in each included study and the PRISMA checklist are shown in Supplementary Tables S1–S2.

**Table 3:** Sample characteristics and prevalence of CLA resistance reported in molecular studies among residents in Santiago, Chile.

Study	Gender female	Median age	Indication for endoscopic examination	Sample size	Prevalence	95% confidence interval
Salinas *et al*.^17^	^___^	^___^	^___^	28	0.46	0.27–0.66
Gonzalez-Hormazabal *et al*.^18^	65%	43	Symptomatic	93	0.31	0.21–0.41
Present study	60%	47	Symptomatic	73	0.26	0.15–0.37
Random effects pooled				194	0.31	0.24–0.39

## Discussion

In 2007, Graham *et al*.^41^ suggested that a first line treatment regime for *H. pylori* was acceptable if eradication rates were superior to 85%. In Latin America, a randomized multicentric eradication trial in 7 countries, including Chile (Santiago), was performed in 2009–2010 to compare STT with sequential therapy for 5 days and sequential therapy for 10 days. STT was the most successful with eradication rates over 85%^42^. In this study, we showed that a 14-day treatment with SST led to a lower eradication rate of 63.4%.

In our study, 26% of the RUT positive patients had 23S mutations compatible with CLA resistance. A2143G mutation was the more frequent, consistent with other international studies^11–13^. In addition, in our meta-analysis a pooled CLA resistance of 31.3% (95% CI 23.9–38.7) was observed, reaffirming a high resistance in Santiago city. On the other hand, Serrano *et al*.^19^ reported high CLA resistance (21%) in pediatric population in Santiago, but this article was excluded from the meta-analysis, because the pooled prevalence was restricted to adult populations. Our multivariable analysis showed that only CLA resistance was significantly and inversely associated with failure of eradication. Several authors have also described lower eradication rates in presence of CLA resistant strains^7,20,43,44^. All the available international guidelines recommend not to use CLA in eradication therapies due to suboptimal eradication rates if CLA resistance is >15%. However, this recommendation should not be generalized for the entire country, due to a heterogeneous CLA resistance reported in other cities of Chile. Supporting this statement, Otth *et al*.^36^ reported in Valdivia (South of Chile) only 9.1% CLA resistance in 2011. A low CLA resistance (6%) was also observed by our group in Curanilahue, a small town in the South of Chile (unpublished data). Nevertheless, a recent study from Temuco (South of Chile) showed a CLA resistance of 40% using agar dilution as antibiotic susceptibility method^40^, which could be related to greater exposure to antibiotics in recent years.

Based on our results, it is necessary to extend the surveillance to antibiotics to other urban and rural populations in our country. A limitation of this study is the low number of subjects, so to give greater consistency to our work, a meta-analysis was performed, but few studies managed to be included not being possible to assess the existence of publication bias. However, the results are consistent with an urban area constantly exposed to the use of antibiotics, which may not be the reality of other regions or rural areas in Chile.

In our study, an eradication rate of 85% was achieved with our second line treatment. Although this treatment showed high prevalence of adverse effects, none of them were severe. A recent meta-analysis by Muñoz N *et al*.^45^, showed that eradication therapy second line treatment achieves over 90% of success with BQT, representing a viable option in high CLA resistance regions like Santiago. As we had described previously, the international guidelines recommend CLA-free regimes such as quadruple therapy with bismuth as the first line, in regions with CLA resistance rates >15%. Other studies have reinforced this recommendation, Zagari RM, *et al*.^46^ in a retrospective multicentre observational study reported “three-in-one” formulation of BQT (capsule containing bismuth subcitrate, tetracycline, and metronidazole) is highly effective and well tolerated.

Nevertheless, in many countries bismuth is not available, so other studies have showed high efficacy with different eradication regimens without bismuth in areas of high CLA resistance. Federico A, *et al*.^47^ reported that a 5-day levofloxacin containing quadruple concomitant therapy is effective and safe. Molina-Infante J, *et al*.^48^ in a multicenter trial showed efficacy of empiric optimized 14-day non-bismuth quadruple therapies (hybrid and concomitant). Tai WC, *et al*.^49^ a randomized controlled study from Taiwan, found a 14-day esomeprazole and amoxicillin containing high-dose dual therapy achieves a high eradication rate as first-line, comparable to that with 7-day non-bismuth quadruple therapy. A recent systematic review and meta-analysis found similar eradication rates for high-dose dual therapy compared to for BQT^50^.

On the other hand, phenotypes derived from the *CYP2C19* polymorphisms found in this study are similar from the data reported by Roco A *et al*. 2012 for healthy Chilean population^51^. Regarding other Latin American populations, Saldaña-Cruz AM *et al*.^52^, reported a proportion of EM of 83%, IM 16.5% and PM 0.2% in a Mexican population, estimates that are similar to other populations in the region, Brazil, Colombia and Bolivia.

In our study, phenotypes of CYP2C19 polymorphisms were not effect on *H. pylori* eradication. This result could be explained by the low number of participants, but also most of the studies that support the association between CYP2C19 polymorphisms and *H. pylori* eradication rates are based on Asian populations, with a greater proportion of poor metabolizers. Nevertheless, large population-based studies should be conducted to determine the clinical impact of CYP2C19 polymorphisms in *H. pylori* eradication in Chile and other high GC risk Latin-American populations.

In conclusion, although our study does not allow a definitive recommendation about first line therapy for *H. pylori* infection in Chile as a country, we found a significant high CLA resistance prevalence associated with *H. pylori* eradication failure in residents in Santiago. Based on these findings and guidelines, we suggest changing STT for CLA free therapies in the urban area of Santiago city. We could recommend BQT or high-dose dual therapy, according to bismuth availability. Further studies need to evaluate the best therapy.

## Supplementary information

Supplementary Tables
